# Spontaneous disappearance and recurrence of impending macular hole: a case report

**DOI:** 10.1186/s13256-019-2277-3

**Published:** 2019-11-17

**Authors:** Makiko Miyamoto, Kazuhiro Shimizu, Yohei Sato, Bunsei Konose, Natsuko Mano, Hiroko Watanabe, Tsunehiko Ikeda

**Affiliations:** 1 Department of Ophthalmology, Takatsuki Hospital, Takatsuki-City, Osaka Japan; 2 Department of Ophthalmology, Osaka Medical College, 2-7 Daigaku-machi, Takatsuki City, Osaka 569-8686 Japan

**Keywords:** Impending macular hole, Spontaneous disappearance, Recurrence, Optical coherence tomography (OCT)

## Abstract

**Background:**

There have been several reports of spontaneous closure and reopening of a macular hole, however, in most of those cases, it was observed in eyes post vitrectomy. Here, we report a case of multiple episodes of spontaneous disappearance and recurrence of impending macular hole (stage 1B macular hole) with no history of previous surgery.

**Case presentation:**

A 76-year-old Japanese man presented with a primary complaint of reduced visual acuity in his right eye. On initial examination, the visual acuity in his right and left eye was 0.4 and 0.01, respectively. He had previously been diagnosed as having macular degeneration of unknown origin in his left eye. Optical coherence tomography imaging confirmed vitreomacular traction and impending macular hole in his right eye. After a 1-week follow-up period, posterior vitreous detachment was detected, and the impending macular hole appeared to be resolved. Two months later, the impending macular hole had completely disappeared and his visual acuity had improved to 0.9. Six months later, he again noticed decreased vision in his right eye. An examination revealed that his visual acuity had dropped to 0.4, and there was a recurrence of impending macular hole. An optical coherence tomography examination showed no definitive findings of vitreous traction, and, 1 month later, spontaneous disappearance was observed again and his visual acuity improved to 0.7.

**Conclusions:**

In this case, both the initial onset and the recurrence involved impending macular hole, however, the optical coherence tomography findings differed at each examination. These findings suggest that some causes other than vitreous traction were responsible for both the spontaneous disappearance and recurrence of the impending macular hole in this present case.

## Background

There have been several reports of spontaneous closures in cases of both idiopathic and traumatic macular holes (MHs) [1, 2]. Some possible mechanisms for the spontaneous closure of an MH are the release of vitreous traction or the proliferation of glial cells. However, the reopening of a MH after a spontaneous closure is rare, and, in most of the previous reports, it occurred following a vitrectomy [3–7]. Many investigators have speculated that the pathogenesis for multiple episodes of spontaneous closure and reopening of a MH is due to tangential traction by the epiretinal membrane (ERM), macular edema, and glial cell proliferation. Here, we report a rare case of an impending MH (IMH) with multiple episodes of spontaneous disappearance and recurrence.

It should be noted that this case is very unusual due to the fact that our patient had no history of previous surgery.

## Case presentation

**Fig. 1:**
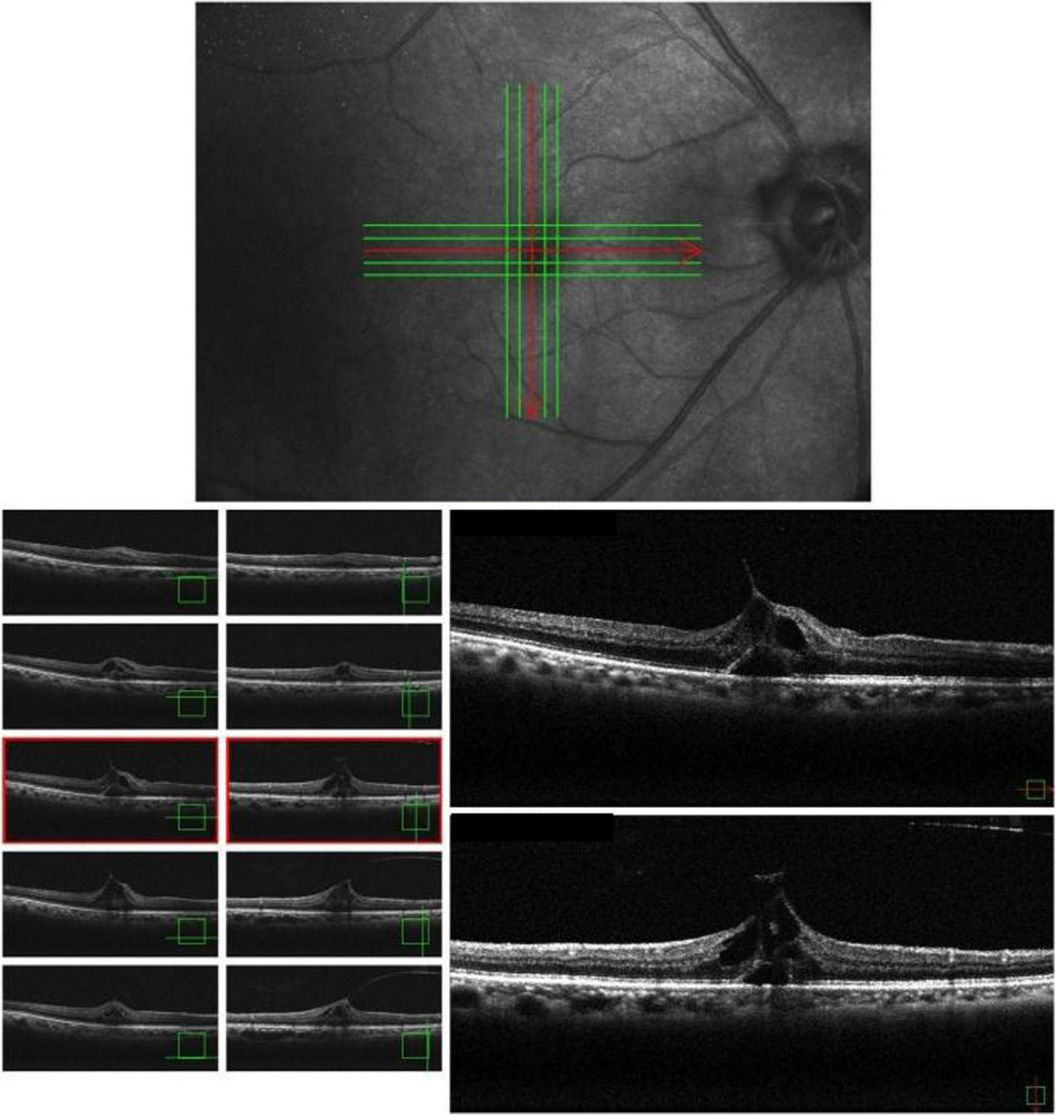
Optical coherence tomography images obtained at the patient’s initial visit showing foveal retinal detachment and cystic formation, and the retinal inner layer continuing in a bridge shape, thus confirming an impending macular hole (stage 1B macular hole)

A 76-year-old Japanese man presented with the primary complaint of decreased visual acuity (VA) in his right eye. Fifty years previously, he had been diagnosed as having macular degeneration of unknown origin in his left eye, yet no particular systemic diseases were found. In February 2017, he became aware of decreased vision in his right eye, and subsequently presented at our hospital on March 16, 2017. He had no particular past medical, social, environmental, family, or employment history of interest. Moreover, he was not receiving any medications and there were no lifestyle-related habits such as tobacco smoking and/or alcohol consumption prior to initial presentation. On examination, his uncorrected VA (UCVA) was 0.4 in his right eye and 0.01in his left eye, and the intraocular pressure in his right and left eyes was 10 mmHg and 11 mmHg, respectively. Both eyes were emmetropic; the axial length in his right eye was 23.62 mm, while that in his left eye was 23.25 mm. There were no abnormal findings in the anterior segment, even though he had a mild cataract in the ocular media. Optical coherence tomography (OCT) imaging revealed vitreous traction in the fovea centralis of his right eye. Moreover, foveal retinal detachment and cystic formation were observed, yet the retinal inner layer continued in a bridge shape, thus signifying IMH (that is, a stage 1B MH) (Fig. 1). In addition, chorioretinal atrophy was observed in his left eye. At the time of admission, there were no abnormalities in the physical and neurological examinations including pulse, blood pressure, and temperature. Laboratory findings, including complete blood count (CBC), liver and renal functions, and urine analysis, also revealed no abnormalities. With the consideration of a possible spontaneous remission, we decided to perform scheduled follow-up examinations.

**Fig. 2:**
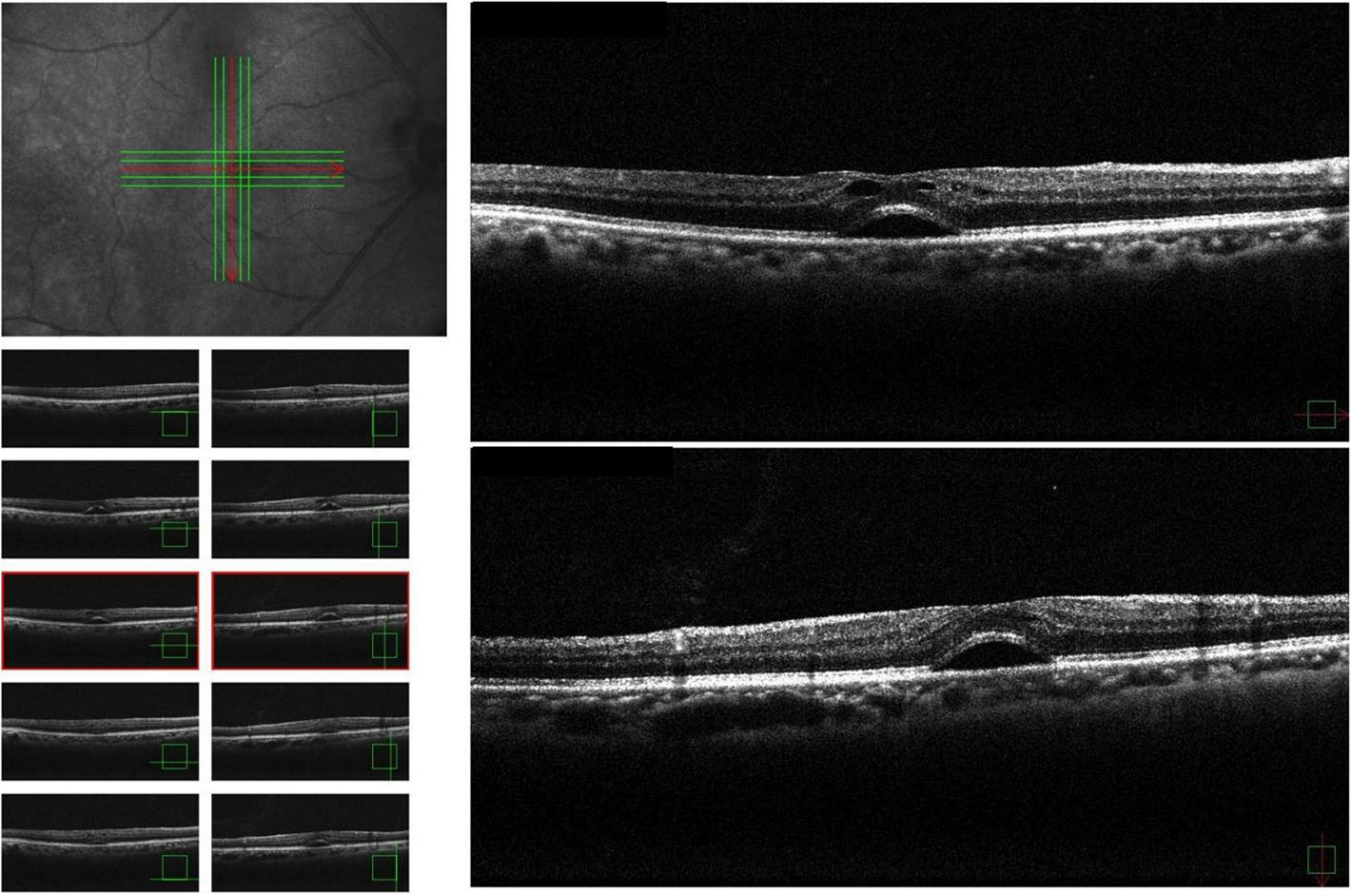
Optical coherence tomography images obtained 1 week after the initial visit showing that the vitreous traction in the fovea centralis had disappeared and that the cystic formation had reduced, leaving only retinal detachment in the fovea centralis

At the initial follow-up examination performed 1 week later, the UCVA in his right eye was found to have improved to 0.6, and an OCT imaging revealed that the vitreous traction in the fovea centralis had disappeared and that the cystic formation had been reduced, leaving only retinal detachment in the fovea centralis (Fig. 2). It should be noted that even though we did not perform fluorescein angiography, there were no obvious abnormalities of the retinal pigment epithelium in his right eye at that time.

**Fig. 3:**
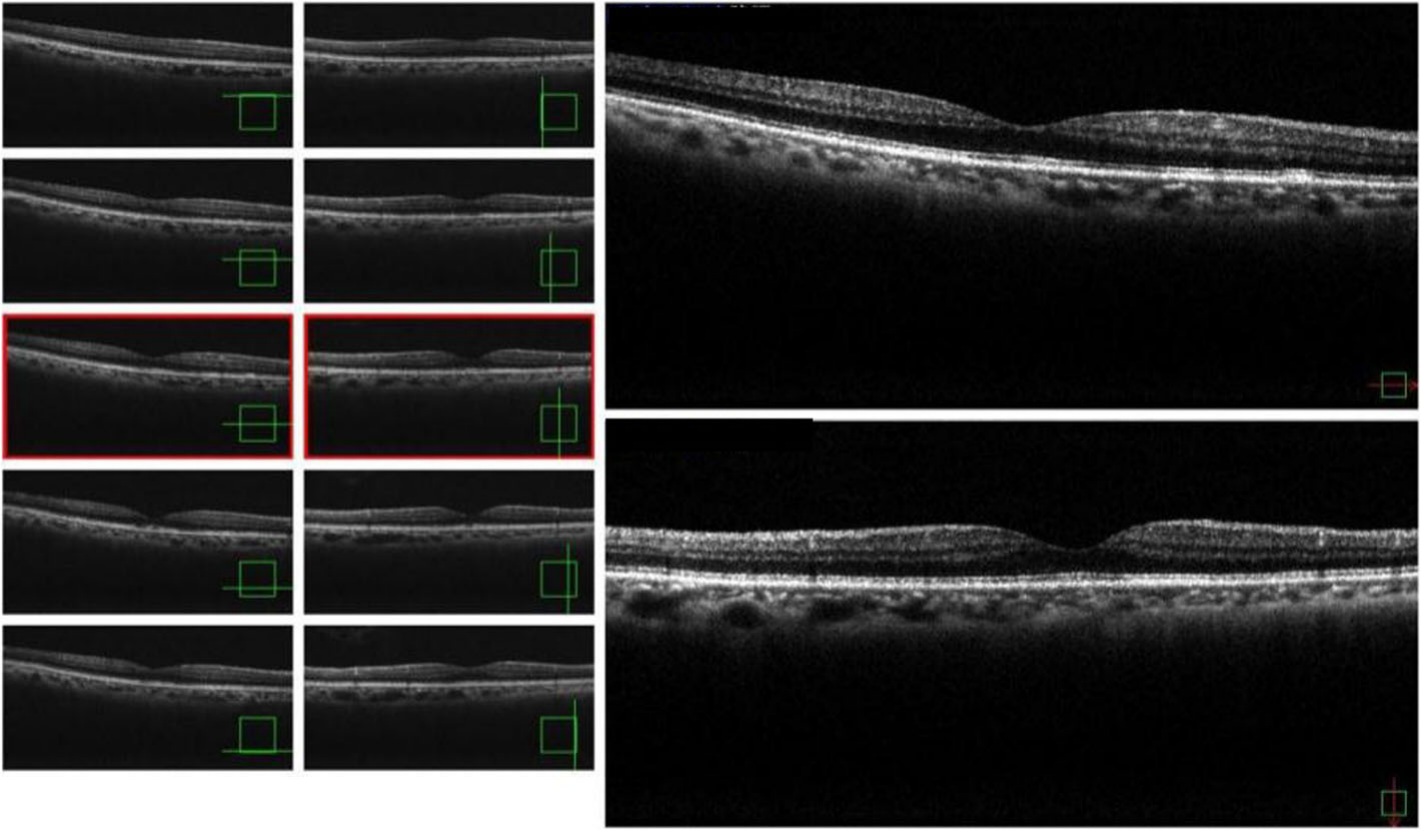
Optical coherence tomography images obtained 2 months after the initial visit showing that retinal detachment in the fovea centralis had resolved

**Fig. 4:**
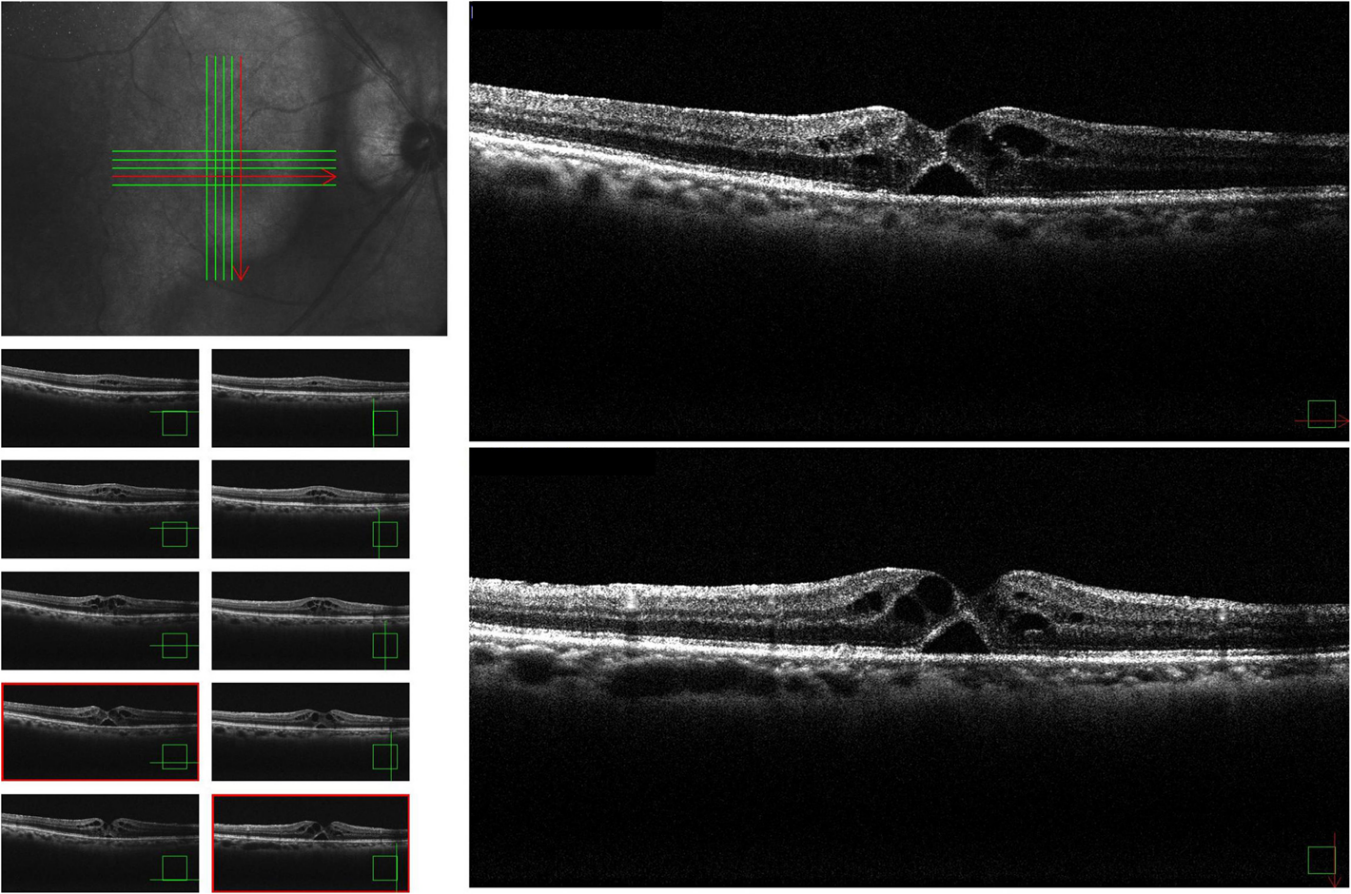
Optical coherence tomography images obtained 6 months after spontaneous closure revealing a recurrence of the impending macular hole

**Fig. 5:**
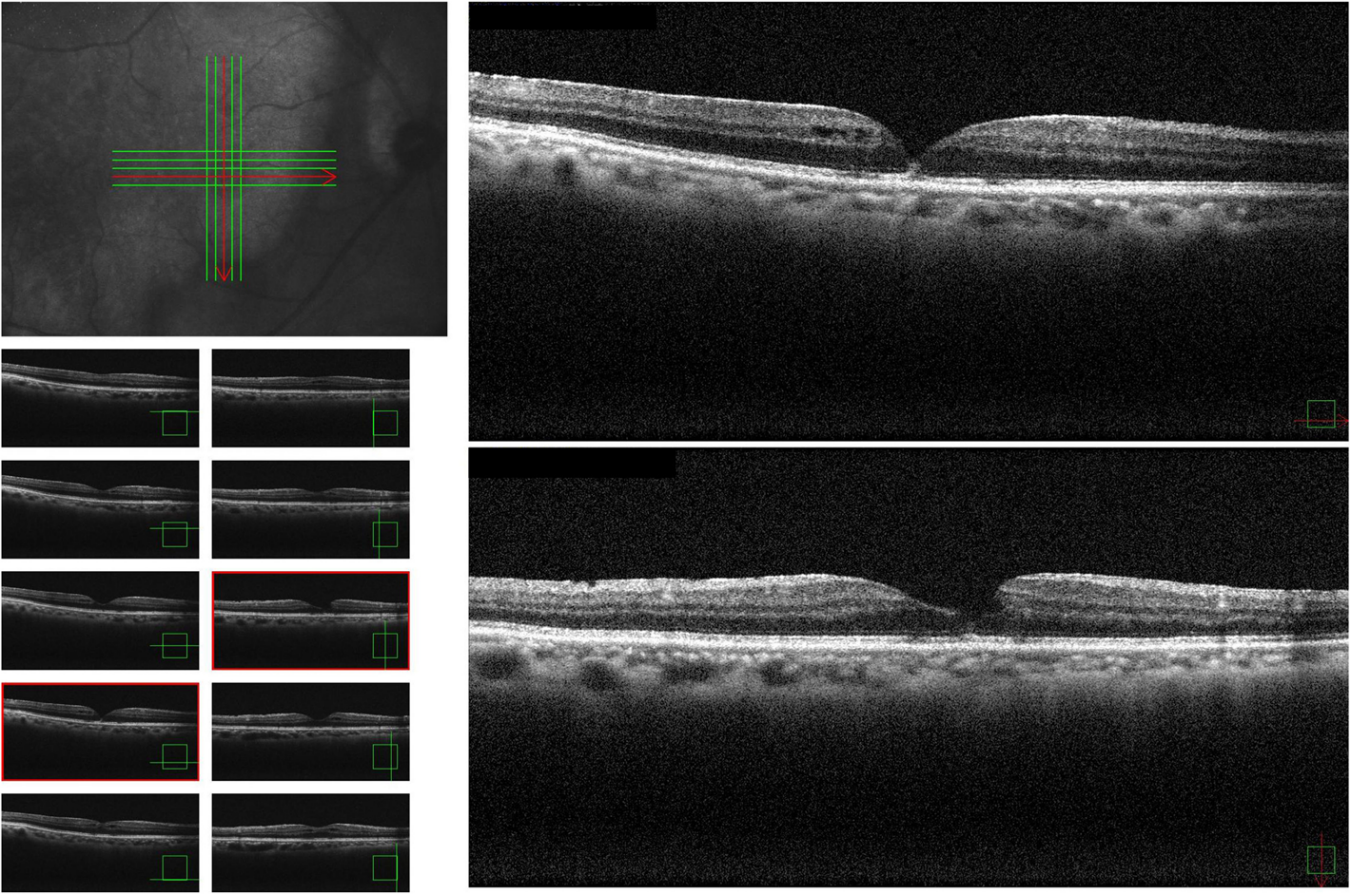
Optical coherence tomography images obtained at approximately 10 months after the spontaneous closure showing improvements in both the cyst formation around the fovea centralis and the fovea retinal detachment

Two months later, his UCVA had improved to 0.9 and the retinal detachment in the fovea centralis had resolved. Moreover, we were able to confirm the line of the inner segment/outer segment (Fig. 3). On November 16, 2017, approximately 6 months post confirmation of the spontaneous remission, he again became aware of decreased VA in his right eye, and returned to our hospital. The UCVA in his right eye had decreased to 0.4 and OCT findings revealed cystic formation around the fovea centralis, as well as a recurrence of the fovea retinal detachment (Fig. 4). Although we speculated an IMH, there was no clear evidence of vitreous traction. We did not perform fluorescein angiography; however, it should be noted that there were still no obvious abnormalities of the retinal pigment epithelium in his right eye. A follow-up examination conducted on December 28, 2017 (1 month later) revealed that the UCVA in his right eye had improved to 0.7. In addition, OCT findings revealed improvements in both the cyst formation around the fovea centralis and the fovea retinal detachment without any treatment (Fig. 5). A follow-up examination performed on February 22, 2018 (2 months later) revealed that the UCVA in his right eye had improved to 0.8.

## Discussion

In this case report, we describe a very unusual and rare case of an IMH with spontaneous closure and reopening with no history of previous surgery. As previously reported by Gass [8], the main cause of MH is thought to be vitreous traction in the fovea centralis. Due to advancements in OCT, ophthalmologists are now able to achieve clearer images of the progression of an MH [9–12]. Kishi *et al.* [13] suggested that due to contraction of the posterior wall of the posterior precortical vitreous pocket, tractional force is applied to the fovea centralis of the retina, which can lead to retinal detachment and fovea centralis cyst formation. Hence, if the traction on the fovea centralis of the precortical vitreous can be relieved, the fovea centralis will repeatedly return to its normal shape.

Similar to the findings above, it has been reported that if the MH has reached stage 2, the completion of posterior vitreous detachment can lead to spontaneous closure in approximately 50% of the cases [1]. In this present case also, we found traction on the fovea centralis during the initial examination, yet, 1 week later, the traction was found to have released and spontaneous remission occurred.

Although the rate of reopening after spontaneous closure in cases of MH is thought to be very low, there have been several previous reports of repeated spontaneous closures and reopenings [3–7]. In the majority of those cases, it reportedly occurred post vitrectomy, and many of those cases involved traction of the ERM over the macula following surgery. Moreover, the primary diseases included rhegmatogenous retinal detachment and diabetic macular edema, which affect the fragility of the macular area.

To date, and to the best of our knowledge, there has only been one previous report of a case of spontaneous closure and reopening of an MH with no history of previous surgery [14]. In that study, the authors reported a case of high myopia with no history of previous surgery in which the spontaneous closure and reopening of the MH occurred three times. In that study, the authors pointed out the involvement of glial cell proliferation as the primary mechanism. The case in this present study has many points in common with that previous case, with the one difference being that our case involved a patient with emmetropia. Although differential diagnoses such as macular edema and serous retinal detachment caused by some other eye disease should be considered, no particular clinical findings were detected in our patient.

It should be noted that it is difficult to attribute the improvement in VA and the restoration of the lamella structure of the fovea centralis simply to glial cell proliferation. In previous studies, we speculated the presence of neural stem cell-like cells with regenerative ability in the fovea centralis [15, 16], and reported the possible involvement of serine proteases such as chymase and tryptase in the vitreous body in the development of MH and ERM [17–19]. Since chymase has an apoptotic effect and tryptase induces tissue fibrosis, we theorized that such serine proteases might be involved in the pathogenesis of MH and ERM. The OCT findings in this present case clearly showed differences in the IMH between the initial occurrence and the subsequent recurrence, thus indicating that different pathogenic mechanisms may be involved. Unfortunately, we were unable to measure the serine proteases in the vitreous body of the case in this present study. However, our assumption is that biochemical factors, in addition to physical factors such as traction, are involved in the spontaneous disappearance and recurrence of the IMH. Further studies are needed to elucidate the pathogenesis of spontaneous disappearance and recurrence of an IMH.

## Conclusions

In this present case, the OCT results revealed an IMH that differed at each examination, that is, at the initial onset and the recurrence, and our findings suggest that some causes other than vitreous traction were responsible for both the spontaneous disappearance and recurrence of the IMH in this patient.
